# Brain tumor segmentation based on deep learning and an attention mechanism using MRI multi-modalities brain images

**DOI:** 10.1038/s41598-021-90428-8

**Published:** 2021-05-25

**Authors:** Ramin Ranjbarzadeh, Abbas Bagherian Kasgari, Saeid Jafarzadeh Ghoushchi, Shokofeh Anari, Maryam Naseri, Malika Bendechache

**Affiliations:** 1 Department of Telecommunications Engineering, Faculty of Engineering, University of Guilan, Rasht, Iran; 2 Faculty of Management and Accounting, Allameh Tabataba‘i University, Tehran, Iran; 3 Faculty of Industrial Engineering, Urmia University of Technology, Urmia, Iran; 4 Department of Accounting, Economic and Financial Sciences, South Tehran Branch Islamic Azad University, Tehran, Iran; 5 Department of Chemical Engineering, Faculty of Engineering, Golestan University, Aliabad Katoul, Iran; 6 School of Computing, Faculty of Engineering and Computing, Dublin City University, Dublin, Ireland

**Keywords:** Cancer, Diseases, Medical research

## Abstract

Brain tumor localization and segmentation from magnetic resonance imaging (MRI) are hard and important tasks for several applications in the field of medical analysis. As each brain imaging modality gives unique and key details related to each part of the tumor, many recent approaches used four modalities T1, T1c, T2, and FLAIR. Although many of them obtained a promising segmentation result on the BRATS 2018 dataset, they suffer from a complex structure that needs more time to train and test. So, in this paper, to obtain a flexible and effective brain tumor segmentation system, first, we propose a preprocessing approach to work only on a small part of the image rather than the whole part of the image. This method leads to a decrease in computing time and overcomes the overfitting problems in a Cascade Deep Learning model. In the second step, as we are dealing with a smaller part of brain images in each slice, a simple and efficient Cascade Convolutional Neural Network (C-ConvNet/C-CNN) is proposed. This C-CNN model mines both local and global features in two different routes. Also, to improve the brain tumor segmentation accuracy compared with the state-of-the-art models, a novel Distance-Wise Attention (DWA) mechanism is introduced. The DWA mechanism considers the effect of the center location of the tumor and the brain inside the model. Comprehensive experiments are conducted on the BRATS 2018 dataset and show that the proposed model obtains competitive results: the proposed method achieves a mean whole tumor, enhancing tumor, and tumor core dice scores of 0.9203, 0.9113 and 0.8726 respectively. Other quantitative and qualitative assessments are presented and discussed.

## Introduction

Brain tumors include the most threatening types of tumors around the world. Glioma, the most common primary brain tumors, occurs due to the carcinogenesis of glial cells in the spinal cord and brain. Glioma is characterized by several histological and malignancy grades, and an average survival time of fewer than 14 months after diagnosis for glioblastoma patients^1^. Magnetic Resonance Imaging (MRI), a popular non-invasive strategy, produces a large and diverse number of tissue contrasts in each imaging modality and has been widely used by medical specialists to diagnose brain tumors^2^. However, the manual segmentation and analysis of structural MRI images of brain tumors is an arduous and time-consuming task which, thus far, can only be accomplished by professional neuroradiologists^3,4^. Therefore, an automatic and robust brain tumor segmentation will have a significant impact on brain tumor diagnosis and treatment. Furthermore, it can also lead to timely diagnosis and treatment of neurological disorders such as Alzheimer’s disease (AD), schizophrenia, and dementia. An automatic technique for Lesion segmentation can support radiologists to deliver key information about the volume, localization, and shape of tumors (including enhancing tumor core regions and whole tumor regions) to make therapy progress more effective and meaningful. There are several differences between the tumor and its normal adjacent tissue (NAT) which hinder the effectiveness of segmentation in medical imaging analysis, e.g., size, bias field (undesirable artifact due to the improper image acquisition), location, and shape^5^. Several models that try to find accurate and efficient boundary curves of brain tumors in medical images have been implemented in the literature. These models can be divided into three main categories:

1. Machine learning approaches address these problems by mainly using hand-crafted features (or pre-defined features)^6^–^9^. As an initial step in this kind of segmentation, the key information is extracted from the input image using some feature extraction algorithm, and then a discriminative model is trained to recognize the tumor from normal tissues. The designed machine learning techniques generally employ hand-crafted features with various classifiers, such as random forest^10^, support vector machine (SVM)^11,12^, fuzzy clustering^3^. The designed methods and features extraction algorithms have to extract features, edge-related details, and other necessary information—which is time-consuming^13^. Moreover, when boundaries between healthy tissues and tumors are fuzzy/vague, these methods demonstrate poorer performances.
2. Multi-atlas registration (MAS) algorithms are based on the registration and label fusion of multiple normal brain atlases to a new image modality^4^. Due to the difficulties in registering normal brain atlases and the need for a large number of atlases, these MAS algorithms have not been successfully dealing with applications that require speed^14^.
3. Deep learning methods extract crucial features automatically. These approaches have yielded outstanding results in various application domains, e.g., pedestrian detection^15,16^, speech recognition and understanding^17,18^, and brain tumor segmentation^19,20^.

Zhang et al.^21^ proposed a TSBTS network (task-structured brain tumor segmentation network) to mimic the physicians’ expertise by exploring both the task-modality structure and the task-task structure. The task-modality structure identifies the dissimilar tumor regions by weighing the dissimilar modality volume data since they reflect diverse pathological features, whereas the task-task structure represents the most distinct area with one part of the tumor and uses it to find another part in its vicinity.

A learning method for representing useful features from the knowledge transition across different modality data employed in^22^. To facilitate the knowledge transition, they used a generative adversarial network (GAN) learning scheme to mine intrinsic patterns from each modality data. Zhou et al.^23^ introduced a One-pass Multi-Task Network (OM-Net) to overcome the problem of imbalanced data in medical brain volume. OM-Net uses shared and task-specific parameters to learn discriminative and joint features. OM-Net is optimized using both learning-based training and online training data transfer approaches. Furthermore, a cross-task guided attention (CGA) module is used to share prediction results between tasks. The extraction of both local and global contextual features simultaneously was proposed inside the Deep CNN structure by Havaei et al.^24^. Their model uses a simple but efficient feature extraction method. An AssemblyNet model was proposed by Coupé et al*.*^25^ which uses the parliamentary decision-making concept for 3D whole-brain MRI segmentation. This parliamentary network is able to solve unseen problems, take complex decisions, and reach a relevant consensus. AssemblyNet employs a majority voting by sharing the knowledge among neighboring U-Nets. This network is able to overcome the problem of limited training data.

Owing to the small size of tumors compared to the rest of the brain, brain imaging data are imbalanced. Due to this characterization, existing networks get to be biased towards the one class that is overrepresented, and training a deep model often leads to low true positive rates. Additionally, existing deep learning approaches have complex structures—which makes them more time-consuming.

To overcome the mentioned difficulties, in our work, a powerful pre-processing strategy to remove a huge amount of unimportant information has been used, which causes promising results even in the present deep learning models. Owing to this strategy, we do not use a complex deep learning model to define the location of the tumor and extract features that lead to a time-consuming process with a high fault rate. Furthermore, thanks to the reduction in the size of the region of interest, the preprocessing step in this strategy also decreases overfitting problems. Besides, after the pre-processing step, a cascade CNN approach is employed to extract both local and global features in an effective way. In order to make our model robust to variation in size and location of the tumor, a new distance-wise attention mechanism is applied inside the CNN model.

This study is structured as follows. In Sect. 2.1, the pre-processing procedure including Z-Score normalization is described in detail for four MRI modalities. In Sect. 2.2, deep learning architecture is described. In Sect. 2.3.1, the distance-wise attention module is demonstrated. In Sect. 2.3.2, the architecture of the proposed Cascade Convolutional Neural Networks (C-ConvNet/C-CNN) is explained. The experiments, discussion, and concluding remarks are in Sects. 3 and 4.

## Material and methods

In this section, we will discuss the proposed method in detail.

### Pre-processing

Unlike many other recent deep learning approaches which use the whole of the image, we only focus on a limited area of it to extract key features. By removing these unnecessary uninformative parts, the true negative results are dramatically decreased. Also, by applying such a strategy, we do not need to use a very deep convolutional model.

#### Similar distributions

**Figure 1:**
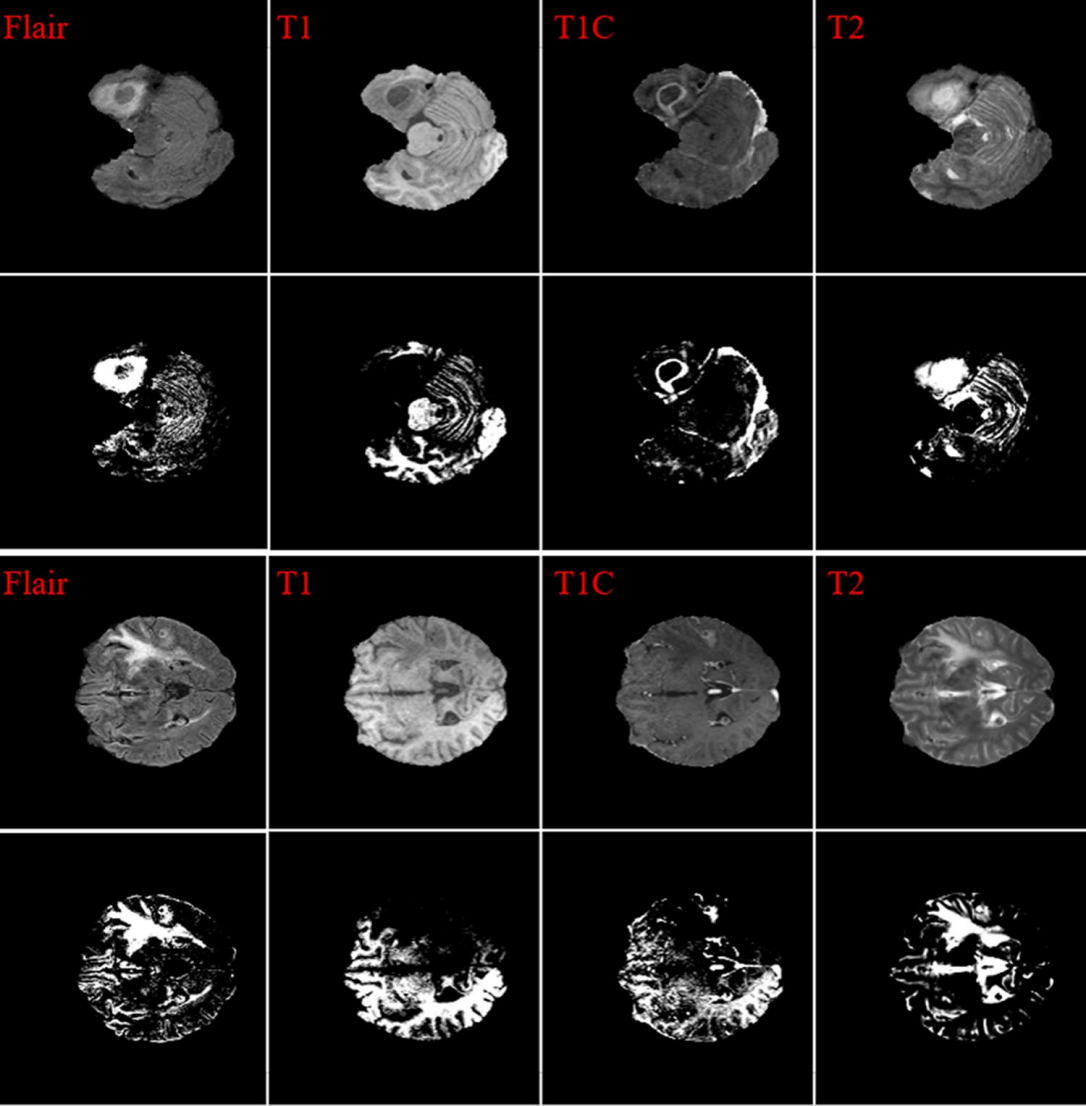
Two sets of four MRI modalities and their corresponding Z-Score normalization.

To improve the final segmentation accuracy, we use four brain modalities, namely T1, FLAIR, T1C, and T2^26,27^. To enforce the MRI data more uniform and remove the effect of the anisotropic (especially for the FLAIR modality), we conduct the Z-Score normalization for the used modalities. By applying this approach to a medical brain image, the output image has zero mean and unit variance^24^. We implemented this step by subtracting the mean and dividing by the standard deviation in only the brain region (not the background). This step was implemented independently for each brain volume of every patient. Figure 1 shows some samples of the four input modalities and their corresponding normalization results.

#### Tumor representation in each slice

**Figure 2:**
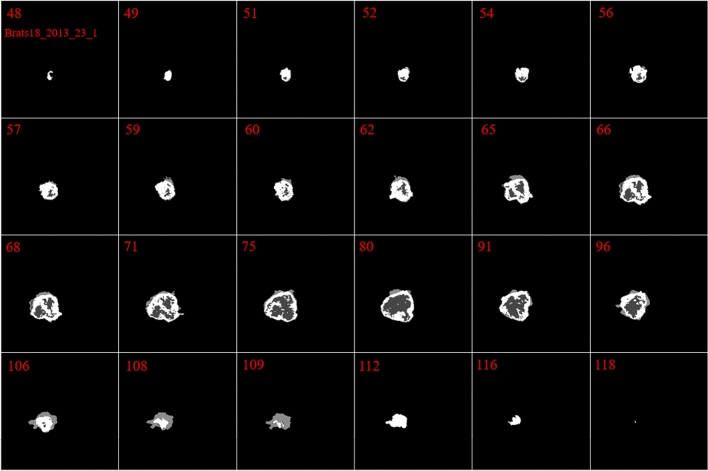
Illustration of the ground truth in 24 different slices in Brats18_2013_23_1. The red numbers indicate the number of the slice.

**Figure 3:**
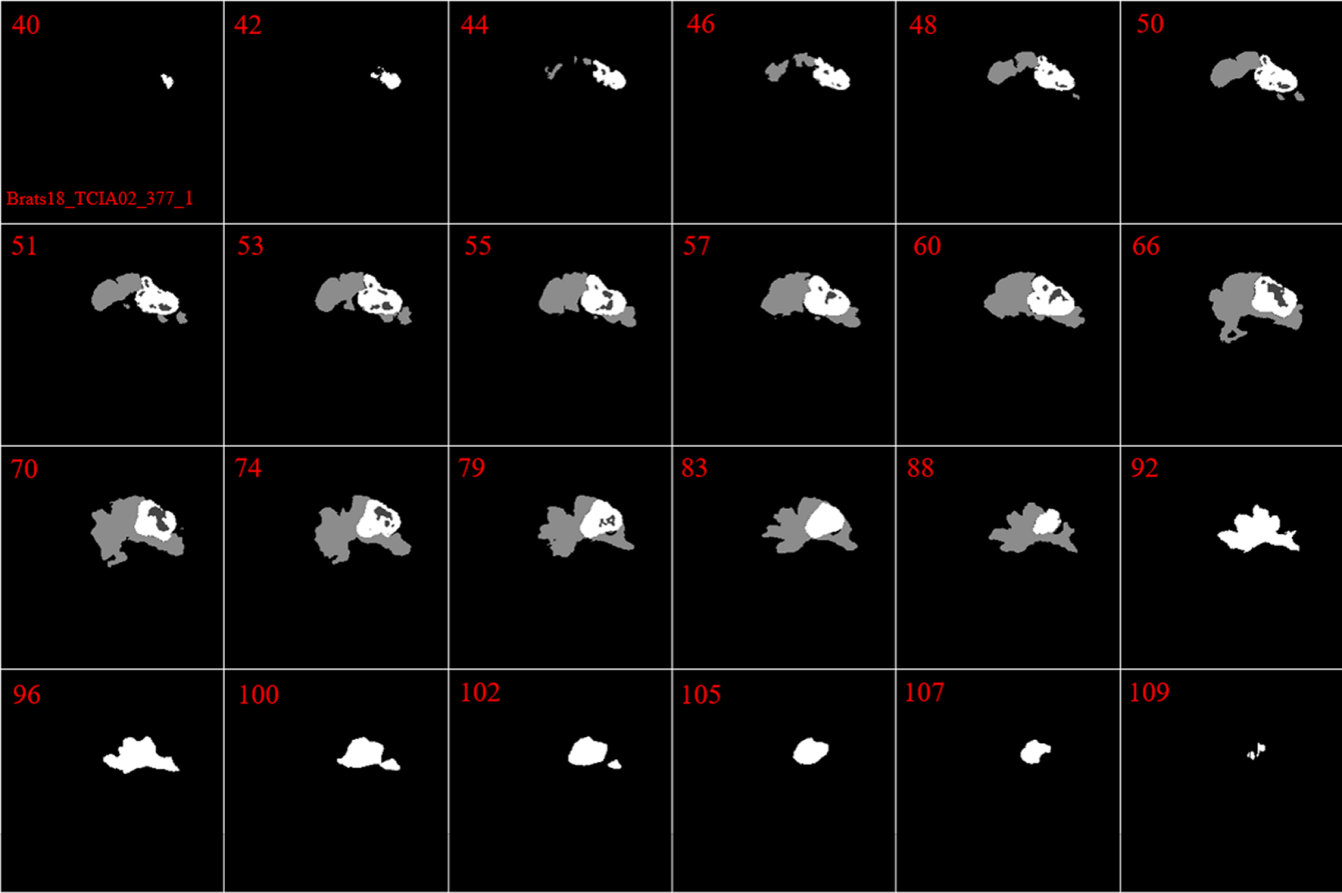
Demonstration of the ground truth in 24 different slices in Brats18_TCIA02_377_1. The red numbers indicate the number of the slice. Different parts of tumor are illustrated with different colors.

In our investigation, we found that the size and the shape of the tumor in sequential slices increase or decrease steadily. The tumor emerges in the first slices with a small size at any possible location of the image. Then, in the following slices, the tumor will remain in the same location inside the image, but it will have a bigger size. Next, after reaching maximum size, the tumor size will start to decrease until it vanishes entirely. This is the core concept of our pre-processing method. These findings are indicated in Figs. 2 and 3.

The main reason for using the mentioned four brain modalities is their unique characteristics for detecting some parts of the tumor. Moreover, to find a tumor, we need to find all three parts in each of the four modalities, then combine them to make a solid object. So, our first goal is to find one part of the tumor in each modality.

#### Finding the expected area of the tumor

**Figure 4:**
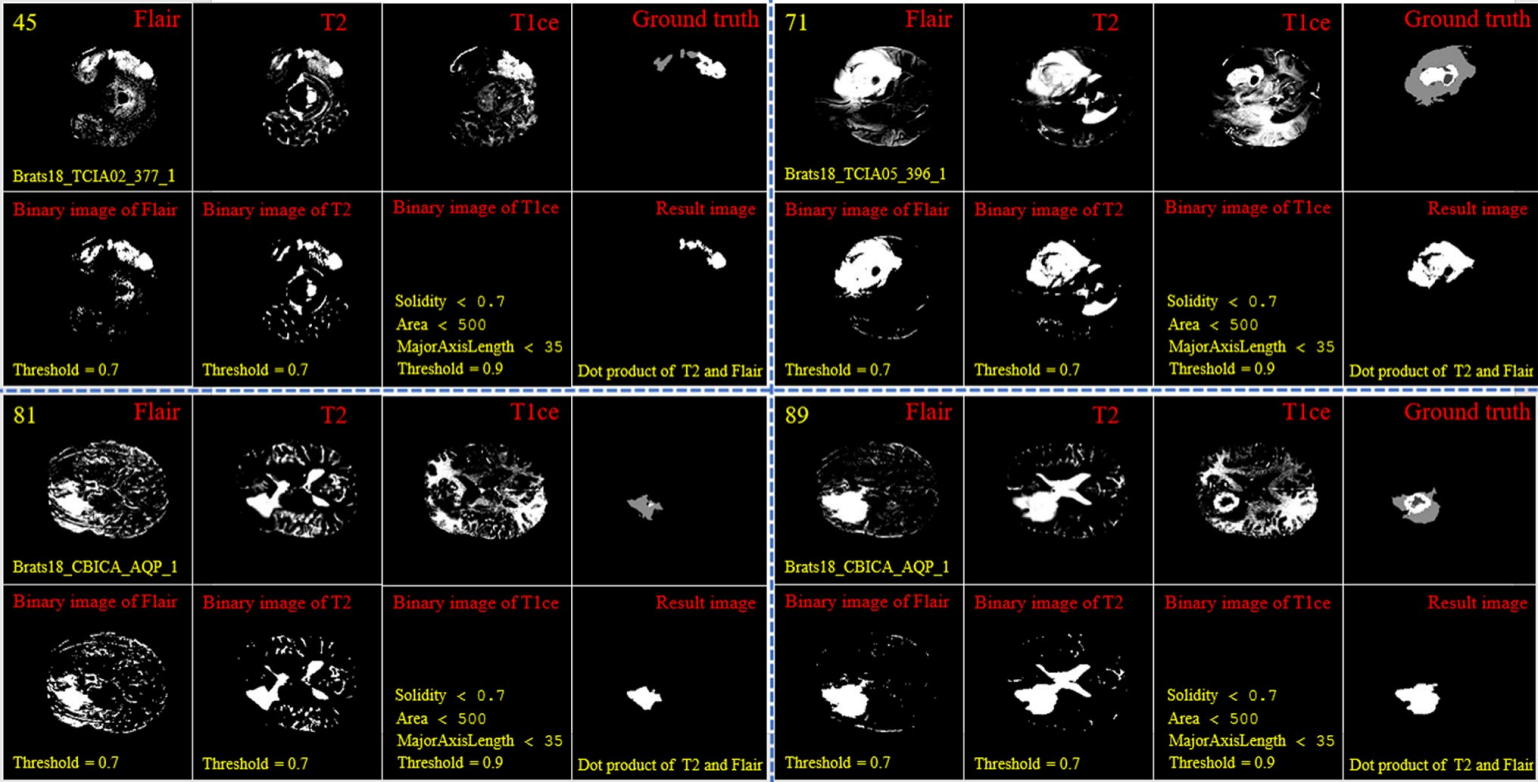
Demonstration of the process of finding a part of the tumor in each slice. The yellow color in the top left corner and the bottom indicates the slice number and sample ID, respectively. Also, the conditions for selecting the object are shown in yellow color. The red color is chosen for identifying the presented image. All binary objects inside the binarized T1ce image are bigger than the threshold criteria, so they were eliminated.

**Figure 5:**
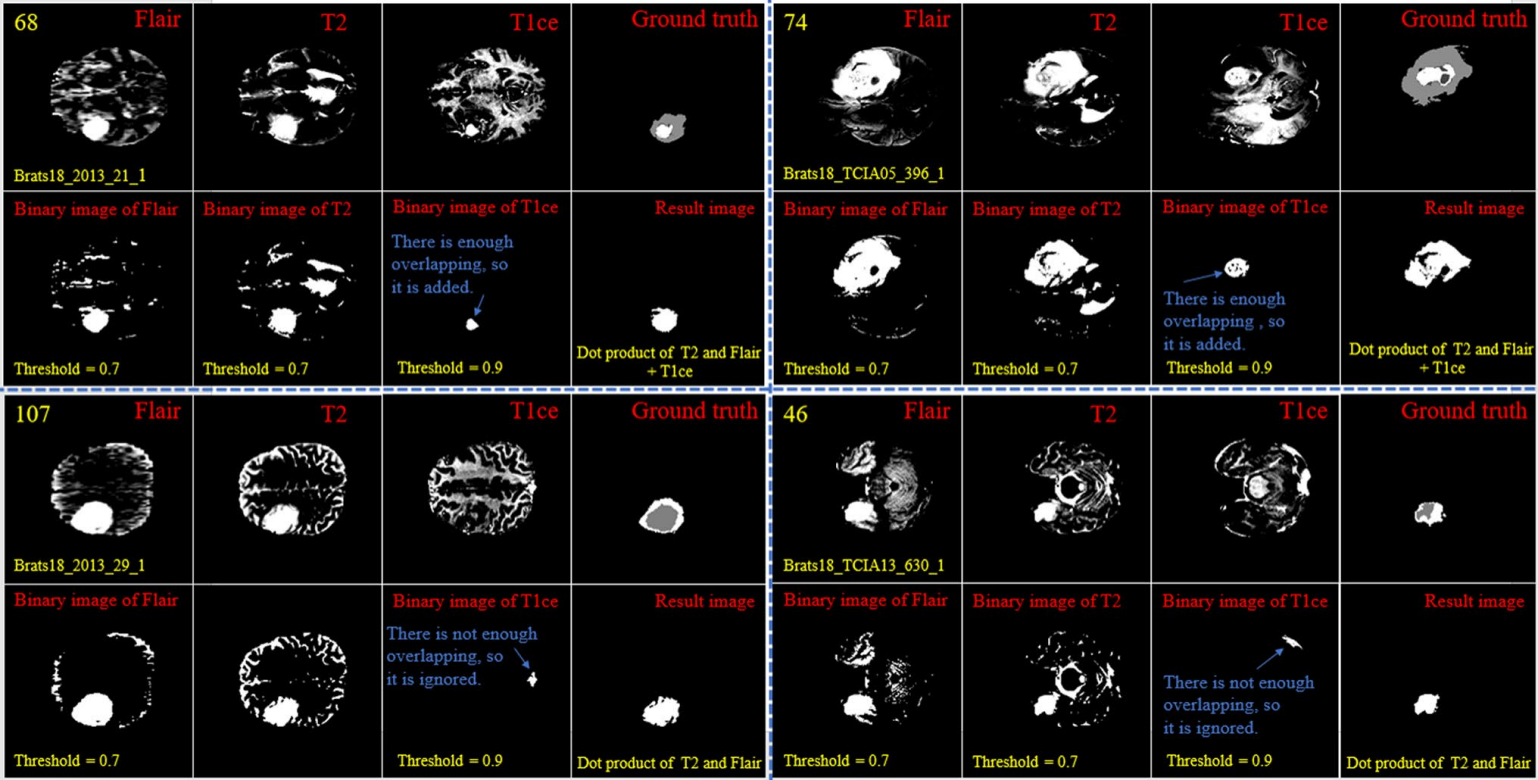
Demonstration of the process of finding a part of the tumor in each slice. The yellow color in the top left corner and the bottom indicates the slice number and sample ID, respectively. Also, the conditions for selecting the object are shown in yellow color. The red color is chosen to identify the presented image. The detected object from T1ce is indicated by the blue text.

By looking deeper into Figs. 2 and 3, we notice emerging, vanishing, and big tumor sizes are encountered in different slices related to different patients. For instance, the biggest tumors are depicted in slices 80 and 74 for Figs. 2 and 3, respectively. Another important fact is that to the best of our knowledge no sharp difference can be observed in the size of continuous slices and tumor size can be varied slightly. During the investigation phase, we noticed that finding the location of the emerging and vanishing tumor is a hard and challenging task. But this is not true when we are looking for the biggest tumor inside the image. To detect the tumor area in each slice we follow four main steps: (1) read all modalities except the T1 image and compute the Z-Score normalized image, (2) binarize the obtained image with the thresholds 0.7, 0.7, and 0.9 for FLAIR, T2, and T1ce, respectively, (3) apply a morphological operator to remove some irrelevant areas, (4) multiply both binary images of FLAIR and T2 to create a new image and 5) combine the obtained areas from each image together. This procedure is demonstrated in Figs. 4 and 5 in details.

As the observed tumor in FLAIR and T2 images is demonstrated with a higher intensity than other parts of the brain, the threshold value of binarization needs to be larger than the mean value (we selected 0.7). Moreover, the tumor is much brighter in T1ce than FLAIR and T2 images. Therefore, a bigger threshold value of binarization needs to be selected (we selected 0.9). If a small threshold value is selected for binarization, several normal tissues will be identified as tumor objects.

In the next step, as there are some tumor-like objects inside the obtained image, we need to discard them using some simple but precise rules. As shown in Figs. 4 and 5, to decide whether to select a binary object as a part of the tumor or not, extra constraints are applied to the binarized T1ce images: (1) object solidity bigger than 0.7, (2) object area bigger than 500 pixels, and (3) length of the major axis of the object needs to be bigger than 35 pixels. Any object in the binarized T1ce image that does not pass these criteria is removed from the image (Fig. 4). The defined constraints (rules) are the same for all the binarized images and we do not need to be altered to obtain good result. Moreover, to overcome the problem of using MRI images with different sizes and thicknesses, the value for each constraint was selected based on a wide span. For instance, in the BRATS 2018 dataset, we defined the smallest object area value as 500 pixels. While using a wide span for selecting an object decreases accuracy, applying the other rules (solidity and major axis length) enables us to overcome that problem effectively.

After detecting all binary objects using morphological operators, we need to add them to each other to create a binary tumor image. But there is still another condition before adding the binarized T1ce to the obtained image from the binary dot product of the FLAIR and T2 images. We can only consider the effect of a binary object inside the T1ce images if it has an overlapping area bigger than 20 pixels with a binary object inside the image obtained from the binary dot product of FLAIR and T2 (Fig. 5).

**Figure 6:**
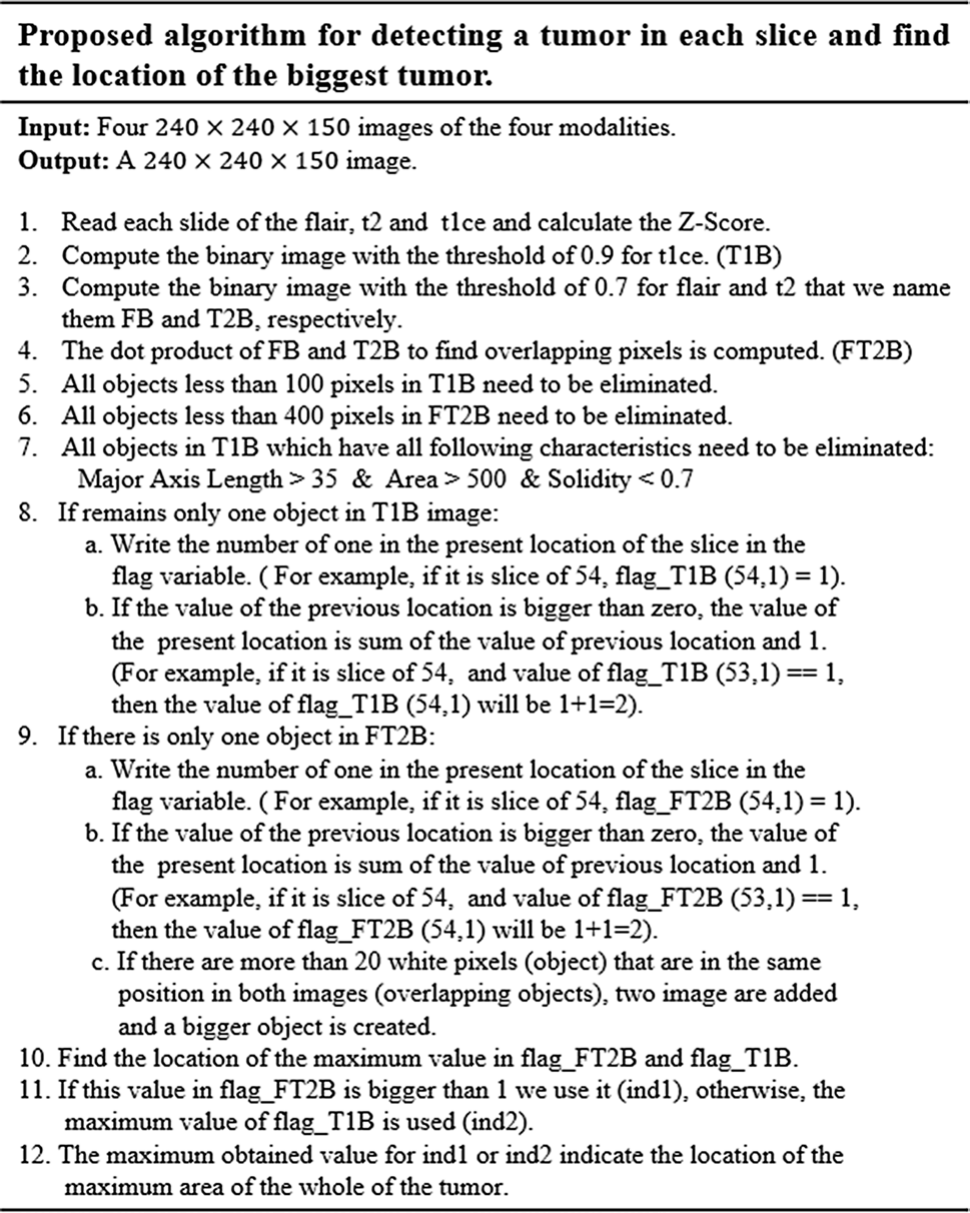
Pseudocode of the proposed algorithm for detecting the biggest tumor among all slices.

**Figure 7:**
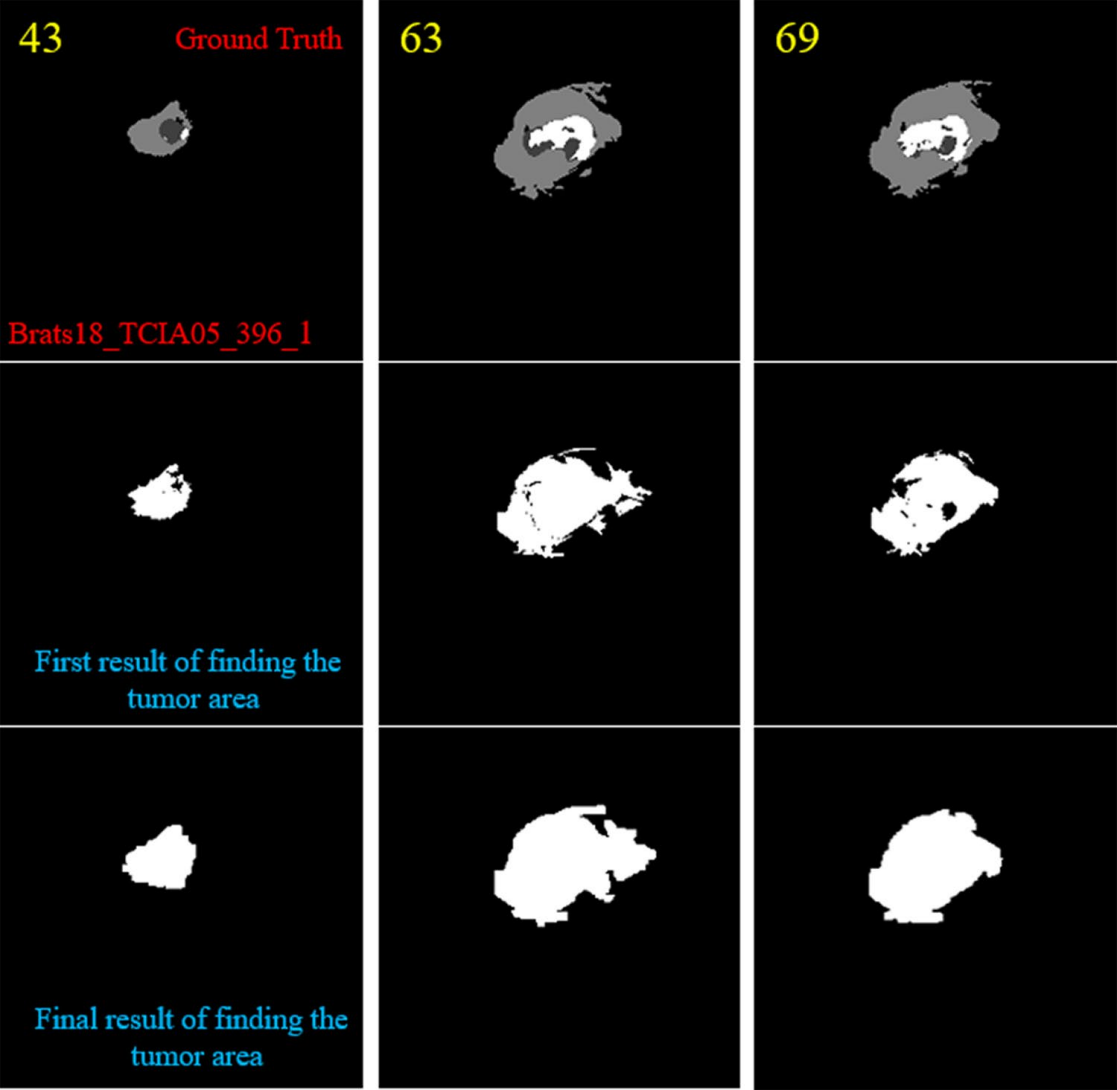
Two examples of finding the tumor object (expected area) and its corresponding center location and applying morphological filters to enlarge the tumor regions. The first row indicates the ground-truth images. The second row demonstrates the tumor object. The third row shows the enlarged tumor objects obtained in the second row. The yellow color in the top left corner indicates the slice number.

In the next step, we need to find the location of the big tumor inside the slices. To this end, we need to be sure that all detected objects are truly tumor objects. To overcome this issue, we track each tumor object in sequential slices. It means if a tumor object is found in almost the same position with a small change in the size in the sequential slices, we can be sure that this object is a true tumor object. After finding the true tumor object in a slice, we search in the same area inside all other slices to find the biggest object. This procedure is explained in Fig. 6 in details. Finally, using morphological operators this object can be enlarged to cover all possible missing tumor areas (we call this area the biggest expected area). By finding this object and its location, we can search only in this area to find the tumor and segment it in all slices (Fig. 7). Finally, based on the information explained in Sect. 2.1.2 and also Figs. 2 and 3, it is obvious that by moving to the first or last slice, the size of the tumor will be decreased. So, we can create a binary mask for all slices in which the size of the expected areas differs slightly from the expected slice to slice difference.

### Deep learning architecture

In today’s artificial intelligence (AI) applications, the convolutional neural network (ConvNet/CNN) pipelines that are a class of deep feed-forward artificial neural networks exhibit a tremendous breakthrough in medical image analysis and processing^28–32^. The structure of a CNN model was inspired by the biological organization of the visual cortex in the human brain which uses the local receptive field. This architecture is similar to that of the connectivity pattern of neurons.

As the CNN model is not invariant to rotation and scale, it is a tremendous task to segment an object that can be moved in the image. One of the key concerns about using a CNN model in the field of medical imaging lies in the time of the evaluation, as many medical applications need prompt responses to minimize the process for additional analysis and treatment. The condition is more complicated when we are dealing with a volumetric medical image. So, by applying a 3D CNN model for detecting lesions using the traditional sliding window approaches, an acceptable result cannot be achieved. This is highly impractical when there are high-resolution volumetric images, and a large number of 3D block samples need to be investigated. In all brain volumetric images, the location, size, orientation, and shape of the tumor are different from a patient to another and cause uncertainty in finding the potential region of the tumor. Also, it is more reasonable to only search a small part of the image rather than the whole image.

To this end, in this work, we first identify the region of interest with a high probability of encountering the tumor and then apply the CNN model to this smaller region–thus reducing computational cost and increasing system efficacy.

The major drawback of convolutional neural network models (CNN) lies in the fuzzy segmentation outcomes and the spatial information reduction caused by the strides of convolutions and pooling operations^32^. To further improve the segmentation accuracy and efficiency, several advanced strategies have been applied to obtain better segmentation results^21,25,33,34^ with approaches like dilated convolution/pooling^35^–^37^, skip connections^38,39^, as well as additional analysis and new post-processing modules like Conditional Random Field (CRF) and Hidden Conditional Random Field (HCRF)^10,40,41^. Using the dilated convolution method causes a large receptive field to be used without applying the pooling layer to the aim of relieving the issue of information loss during the training phase. The skip connection has the capability of restoring the unchanged spatial resolution progressively with the integration of features and adding outputs from previous layers to the existing layer in the down-sampling step.

Recently, the attention mechanism has been employed in the deep learning context that has shown excellent performance for numerous computer vision tasks including instance segmentation^42^, image-denoising^43^, person re-identification^44^, image classification^45,46^, etc.

### Proposed structure

In this study, a cascade CNN model has been proposed that combines both local and global information from across different MRI modalities. Also, a distance-wise attention mechanism is proposed to consider the effect of the brain tumor location in four input modalities. This distance-wise attention mechanism successfully applies the key location feature of the image to the fully-connected layer to overcome overfitting problems using many parallel convolutional layers to differentiate between classes like the self-co-attention mechanism^47^. Although many CNN-based networks have been employed for similar multi-modality tumor segmentation in prior studies, none of them uses a combination of an attention-based mechanism and an area-expected approach.

#### Distance-wise attention (DWA) module

**Figure 8:**
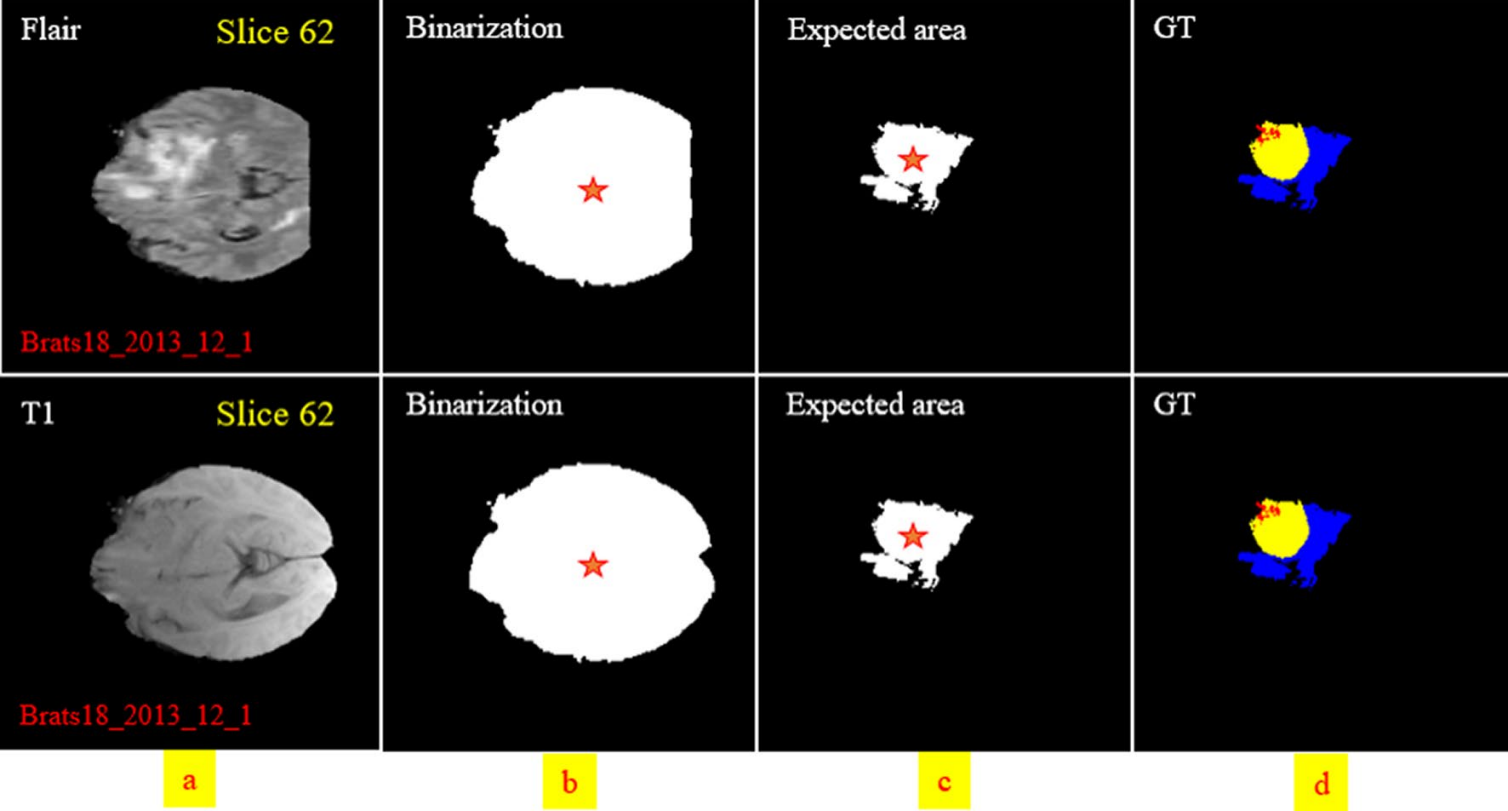
An example depicting the whole brain and its corresponding binary mask for two modalities. The expected area is shown in the third column. The center of the binary mask and the expected area is shown by a red star.

By considering the effect of dissimilarity between the center of the tumor and the expected area, we can guess the probability of encountering each pixel in the investigating process. In other words, knowing the location of the center of the expected (see Fig. 8) leads to a better differentiation between pixels of the three tumor classes.

**Figure 9:**
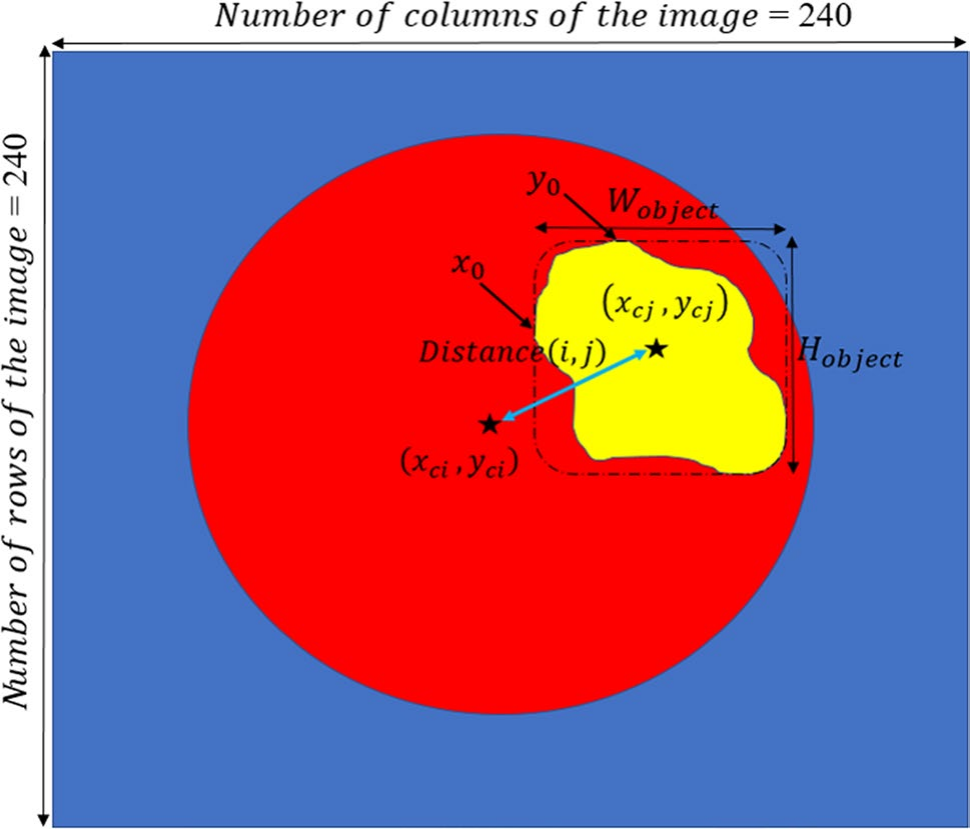
Illustration of parameter calculation in the Distance-Wise Attention (DAW) module. The blue and red pixels are the background and the brain, respectively. The expected area is represented by a yellow object. The size of the image is 240 $\times$ 240.

The DWA module explores distance-wise dependencies in each slice of the four employed modalities for the selection of useful features. Given an input feature channel set ${\mathbb{A}}\in {\mathbb{R}}^{H\times W\times N}$, ${\mathbb{A}} = \left\{ {{\mathbb{A}}_{1} ,{\mathbb{A}}_{2} , \ldots , {\mathbb{A}}_{N} } \right\}$, where ${\mathbb{A}}_{i} \in {\mathbb{R}}^{H \times W}$ indicates a channel. The variables N, H, and W, are the input channels, spatial height, and spatial width, respectively. So, as it is shown in Fig. 9, the $O^{th}$ centroid of the object is obtained on each channel map by

1 $$\left\{ {\begin{array}{*{20}c} {y_{c} = y_{0} + \frac{{H_{{{\text{object}}}} }}{2}} \\ {x_{c} = x_{0} + \frac{{W_{{{\text{object}}}} }}{2}} \\ \end{array} } \right.$$

2 $$\left\{ {\begin{array}{*{20}c} {W_{{{\text{object}}}} = \max \,\arg \left( {{\text{sum}}\,{\text{pixels}} = = 1 {\text{in}}\,{\text{each }}\,{\text{ow}}} \right) } \\ {H_{{{\text{object}}}} = \max \,\arg \left( {{\text{sum}}\,{\text{pixels}} = = 1 {\text{in }}\,{\text{each}}\,{\text{column}}} \right)} \\ \end{array} } \right.$$

3 $$\left\{ {\begin{array}{*{20}c} {y_{0} = {\text{location }}\,{\text{of}}\,{\text{the }}\,{\text{starting}}\,{\text{point}}\,{\text{in}}\,H_{{{\text{object}}}} } \\ {x_{0} = {\text{location}}\,{\text{of}}\,{\text{the }}\,{\text{starting}}\,{\text{point}}\,{\text{in}}\,W_{{{\text{object}}}} } \\ \end{array} } \right.$$

where $y_{c}$ and $x_{c}$ represent the center of the white object, $W_{object}$ and $H_{object}$ indicate the width and height of the object, respectively.

**Figure 10:**
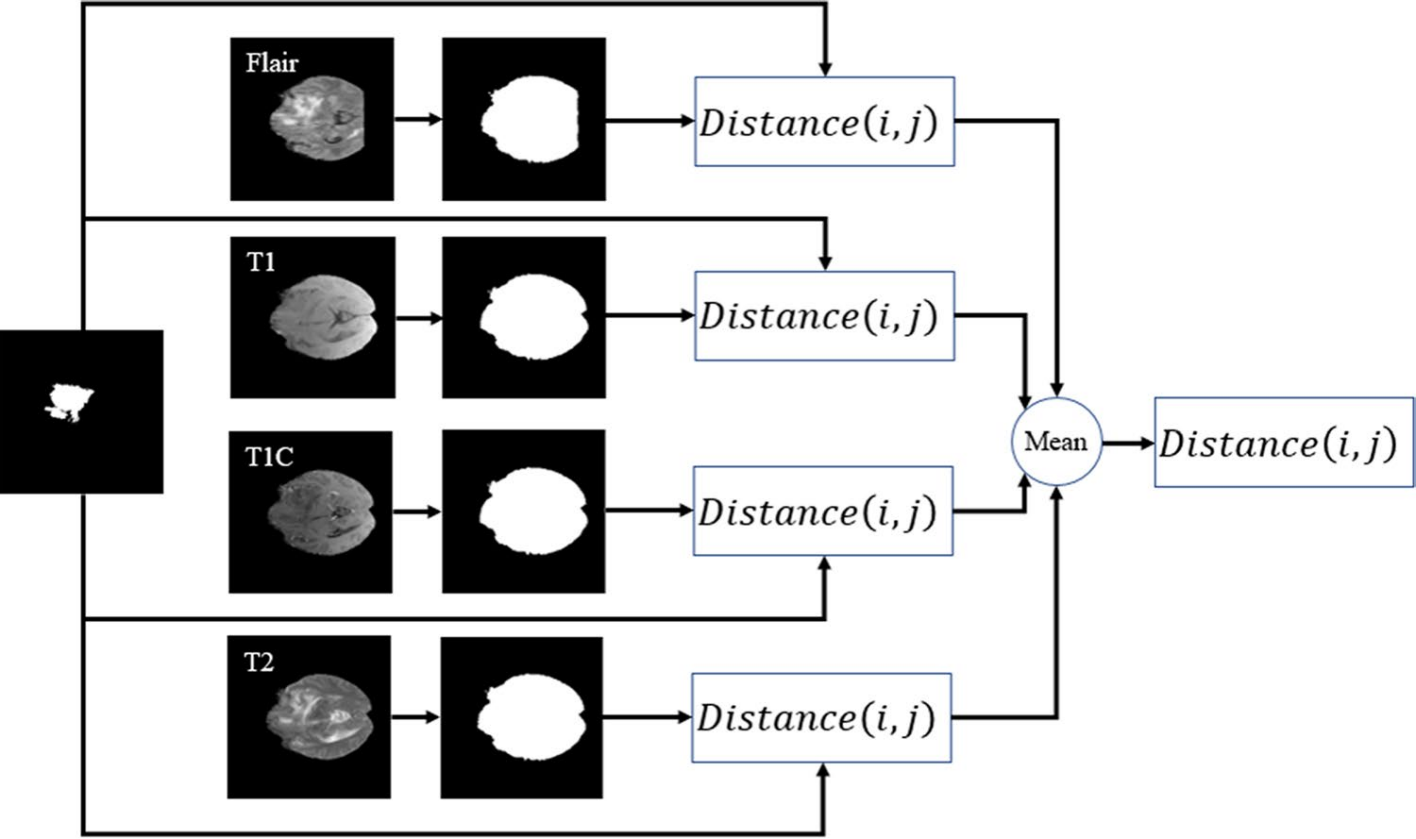
Distance calculation based on the center of the expected area and the four input modalities mask.

By calculating Eq. (1) for both the expected area (see Fig. 8c) and binarization of the input modality in each slide (see Fig. 8b), the distance-wise can be defined as

4 $${\text{Distance}}\left( {i,j} \right) = \frac{{\sqrt[2]{{\left( {x_{ci} - x_{cj} } \right)^{2} + \left( {y_{ci} - y_{cj} } \right)^{2} }}}}{{{\text{Number}}\,{\text{of}}\,{\text{rows}}\,{\text{of}}\,{\text{the}}\,{\text{image}}}}$$

where i and j represent the binarized input modality and expected region, respectively. To obtain the width $W_{object}$ of the object in Eq. (2), we need to count the number of pixels in each row that have the value 1, and then select the row with the maximum count. For calculating the height ${H}_{object}$, we do the same strategy but in vertical. Figure 9 provides more details about computing parameters in the DAW module. As shown in Fig. 10, this process is done for all input modalities and the mean of them is fed to the output of the module for each slice.

#### Cascade CNN model

**Figure 11:**
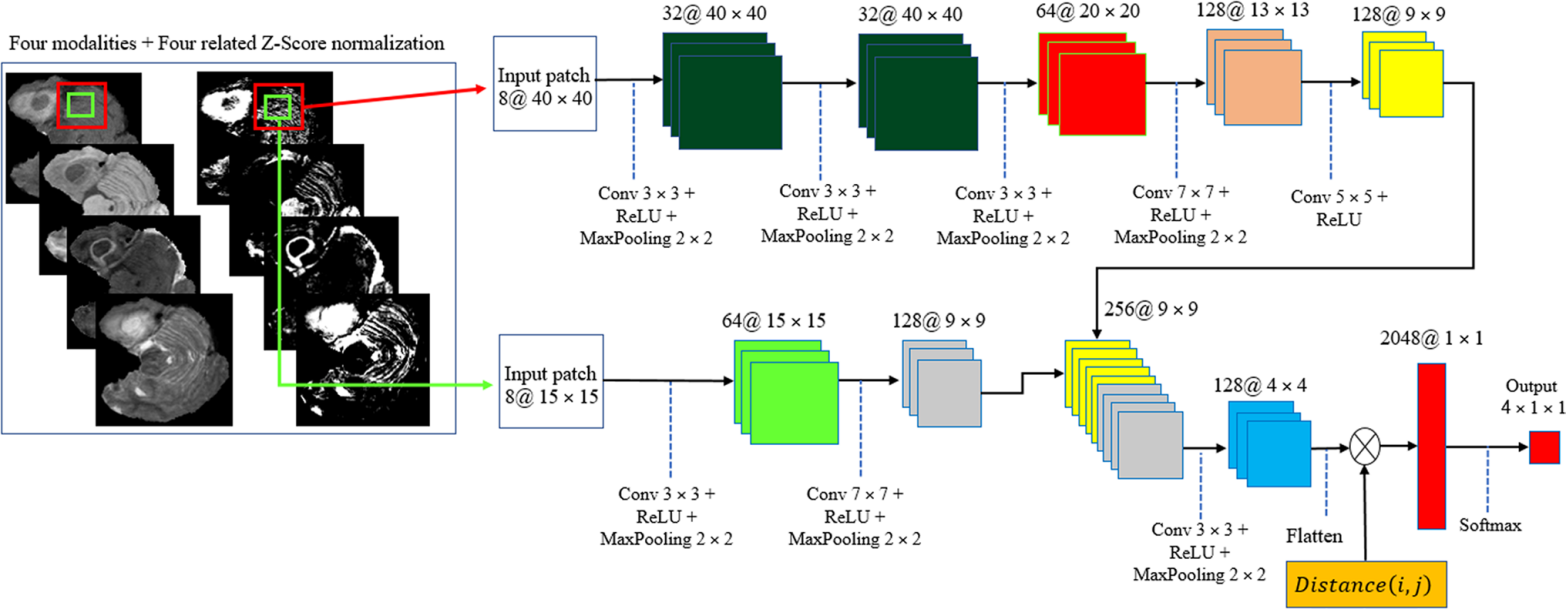
Our implemented cascade structure. The green and red windows inside the input images represent the local and global patches, respectively. The DWA module is represented at the end of the structure before the FC layer.

The flowchart of our cascade mode is depicted in Fig. 11. To capture as many rich tumor features as possible, we use four modalities, namely, fluid attenuated inversion recovery (Flair), T1-contrasted (T1C), T1-weighted (T1), T2-weighted (T2). Moreover, we add four corresponding Z-Score normalized images of the four input modalities to improve the dice score of segmentation results without adding more complicated layers to our structure.

Due to the use of a powerful preprocessing step that eliminates about 80% of the insignificant information of each input image, there is no need for a complex deep network such as^10,22,32^. In other words, by selecting approximately 20% of the whole image (this percentage is the mean of the whole slices of a patient) for each input modality and corresponding Z-Score normalized image, there fewer pixels to investigate.

Also, considering the effect of the center of the tumor to correct detection leads to improve the segmentation result without using a deep CNN model. So, in this study, a cascade CNN model with eight input images is proposed which employs the DWA module at the end of the network to avoid overfitting.

**Figure 12:**
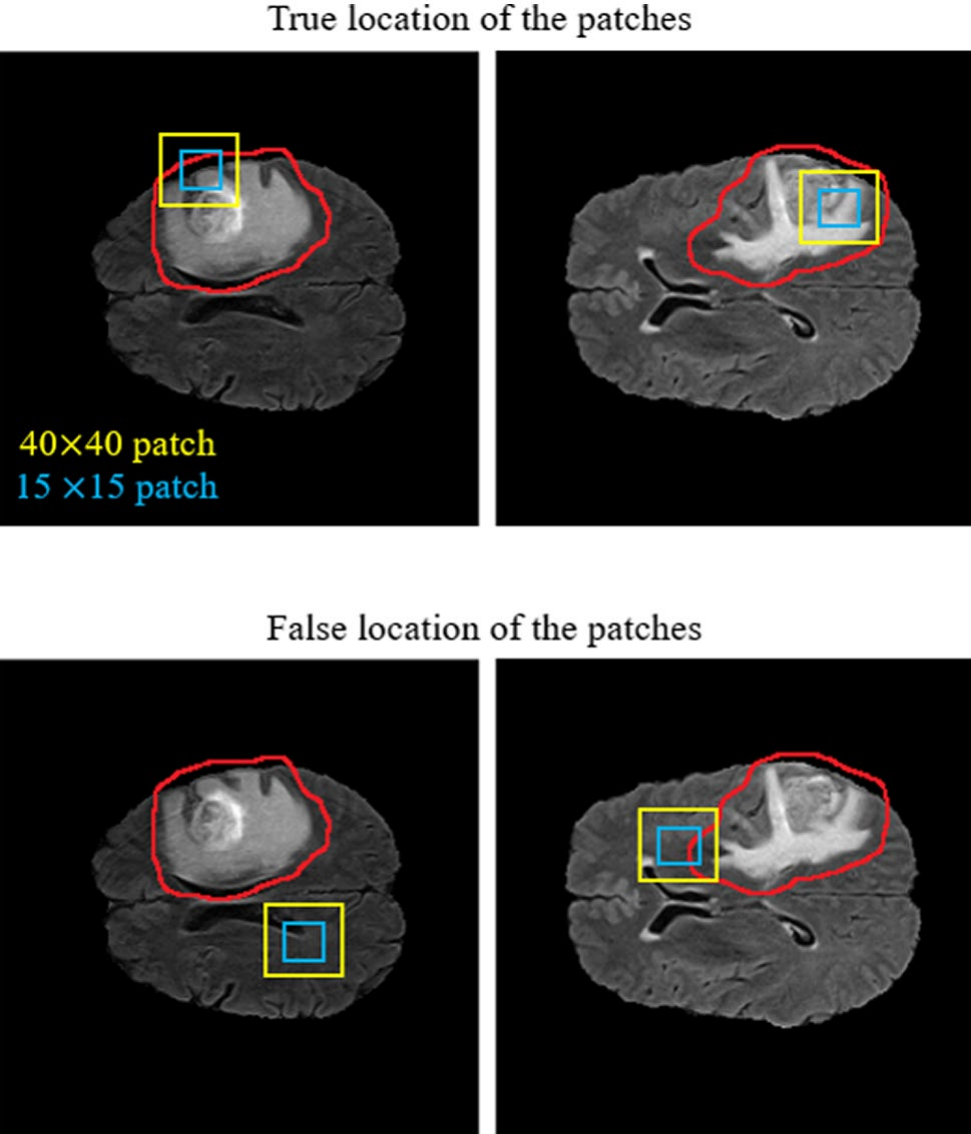
Our implemented cascade structure. The blue and yellow windows inside the input images represent the local and global patches, respectively. The red contour indicates the obtained expected area.

As demonstrated in Fig. 11, our CNN model includes two different routes which extract local and global features from the four input modalities and the corresponding Z-Score normalized images. The key goal of using the first route is detecting the pixels on the border of each tumor (the global feature), whereas the key goal of the second route is labelling each pixel inside the tumor (the local feature). In the first route, a 40 × 40 patch (red window) is selected from each input image to feed the network. It is worth noting that we extract only patches that have their centers located in the obtained expected area, as shown in Fig. 12. The presence of Z-Score normalized images improves the accuracy of the tumor border recognition. The number of convolutional layers for extracting the global feature is five. Unlike the first route, in the local feature extraction route, there are only two convolution layers and they are both fed with eight 15 × 15 input patches (green window). The core building block of the proposed CNN structure is expressed as the convolutional layer. This layer can calculate the dot-product between input data with arbitrary size and a set of learnable filters (masks), much like a traditional neural network^32,48,49^.

The size of the applied masks is always smaller than the dimensions of the input data in all kinds of CNNs. Regularly, the first convolution layers which are applied at the beginning of the CNN model play a significant role in extracting low-level features such as luminance and texture discontinuity^50,51^. The high-level features including tumor region masks are investigated in the deeper convolutional layers of the pipeline, while the middle convolutional layers are utilized for investigating the mid-level features including edges, curves, and points.

As demonstrated in the first row of Fig. 12, the center of each patch is located inside the red border, regardless of whether there is part of the window outside the red border or not. By doing this, we do not investigate insignificant areas (which do not include the tumor). This is more helpful and reasonable when we are encountering imbalanced data. So, samples of the lesion are being equalized to the normal tissue which avoids overfitting in the training step. Additionally, this approach is helpful when dealing with images of various sizes and thicknesses as insignificant parts of the images are discarded before affecting the recognition of the tumor algorithm.

After each convolution layer, there is an activation layer that helps the network to learn complex patterns without changing the dimension of the input feature maps^52^. In other words, in the case of an increased number of layers and to overcome the vanishing gradient problem in the training step, an activation function is applied to each feature map to enhance the computational effectiveness by inducing sparsity^51,53^.

In this study, all negative values are changed to zero using the Non-Linearity (ReLU) activation function which acts as a linear function for positive and zero values. It means some nodes obtain null weights and become useless and do not learn anything. So, fewer neurons would be activated because of the limitations applied by this layer.

In contrast to the convolution operation, the pooling layer which is regularly incorporated between two sequential convolutional layers has no parameters and summarizes the key information without losing any details in the sliding window (mask). Additionally, as the dimension of the feature maps (in both column and row) is decreased in this layer, the training time will be smaller and mitigates overfitting^32,49^. By using the max-pooling method in this paper, the feature map is divided into a set of regions with no overlapping, then takes the maximum number inside each area.

As in a CNN pipeline, the dimension of the receptive field does not cover the entire spatial dimension of the image in the last convolutional layer, the produced maps by the last convolutional layer related to only an area of the whole input image. Due to this characterization of the receptive field, to learn the non-linear combinations of the high-level features, one or more FC layers have to be used. It should be noticed that before employing the achieved feature maps in the fully connected layer, these two-dimensional feature maps need to be changed into a one-dimensional matrix^54^. Furthermore, to reduce the effect of the overfitting a dropout layer^55^ with a 7% dropout probability has been employed (before the FC layer).

Unlike the convolutional layers, the fully connected layers are composed of independent more parameters, so they are harder to train^56^. The last layer in the proposed pipeline for the classification task is the Softmax regression (Multi-class Logistic Regression) layer that is used to distinguish one class from the others. This Multi-class Logistic regression can follow a probability distribution between the range [0,1] by normalizing an input value into a vector of values. This procedure demonstrates how likely the input data (image) belongs to a predefined class. It should be mentioned that the sum of the output probability distribution is equal to one^24,48^.

In the proposed network, we employed the stochastic gradient descent approach as the cross-entropy loss function to overcome the class imbalance problem^57^. This loss function calculates the discrepancy between the ground truth and the network’s predicted output. Also, in the output layer, four logistic units were utilized to investigate the probabilities of the given sample belonging to either of the four classes. The loss function can be formulated as follows:

5 $$loss_{i} = - \log \left( {\frac{{e^{{U_{p} }} }}{{\mathop \sum \nolimits_{d = 1}^{Q} e^{{U_{d} }} }}} \right)$$

where $loss_{i}$ implies the loss for the i-th training sample. Also, $U_{p}$ demonstrates the unnormalized score for the ground-truth class P. This score can be generated by considering the effect of the outputs of the former FC layer (multiplying) with the parameters of the corresponding logistic unit. To get a normalized score to determine the between-class variation in the range of 0 and 3, the denominator adds the predicted scores for all the logistic units Q. As only four output neurons have been used in this study, the value for Q is equal to four. In other words, each pixel can be categorized into one of four classes.

## Experiments

### Data and implementation details

In this study, training, validation, and testing of our pipeline have been accomplished on the BRATS 2018 dataset which includes the Multi-Modal MRI images and patient’s clinical data with various heterogeneous histological sub-regions, different degrees of aggressiveness, and variable prognosis. These Multi-Modal MR images have the dimensions of $240\times 240\times 150$ and were clinically obtained using various magnetic field strengths, scanners, and different protocols from many institutions that are dissimilar to the Computed Tomography (CT) images. There are four MRI sequences for training, validation, and testing steps which include the Fluid Attenuated Inversion Recovery (FLAIR), highlights water locations (T2 or T2-weighted), T1 with gadolinium-enhancing contrast, and highlights fat locations (T1 or T1-weighted).

This dataset includes 75 cases with LGG and 210 cases with HGG which we randomly divided into training data (80%), validation data (10%), and test data (10%). Also, labels of images were annotated by neuro-radiologists with tumor labels (necrosis, edema, non-enhancing tumor, and enhancing tumor are represented by 1, 2, 3, and 4, respectively. Also, the zero value indicates a normal tissue). Label 3 is not used.

The experimental outcomes are achieved for the proposed structure using MATLAB on Intel Core I7- 3.4 GHz, 32 GB RAM, 15 MB Cache, over CUDA 9.0, CuDNN 5.1, and GPU 1080Ti NVIDIA computer under a 64-bit operating system. We adopted the Adaptive Moment Estimation (Adam) for the training step, with a batch size 2, weight decay 10^−5^, an initial learning rate 10^−4^. We took in total 13 h to train and 7 s per volume to test.

### Evaluation measure

The effectiveness of the approach is assessed by metrics regarding the enhancing core (EC), tumor core (TC, including necrotic core plus non-enhancing core), and whole tumor (WT, including all classes of tumor structures). The Dice similarity coefficient (DSC) is employed as the evaluation metric to compute the overlap between the ground truth and the predictions.

The experimental results were obtained using the three criteria, namely HAUSDORFF99, Dice similarity, and Sensitivity^23,58–60^. The Hausdorff score assesses the distance between the surface of the predicted regions and that of the ground-truth regions. Dice score is employed as the evaluation metric for computing the overlap between the ground truths and the predictions. Specificity (actual negative rate) is the measure of non-tumor pixels that have been calculated correctly. Sensitivity (Recall or True positive rate) is the measure of tumor pixels that have been correctly calculated. These three criteria can be formulated as:

6 $${\text{DICE}}\left( {{\text{R}}_{{\text{p}}} ,{\text{R}}_{{\text{a}}} } \right) = 2{*}\frac{{{\text{R}}_{{\text{p}}} \cap {\text{R}}_{{\text{a}}} }}{{{\text{R}}_{{\text{p}}} + {\text{R}}_{{\text{a}}} }}{ }$$

7 $${\text{Sensitivity}} = \left( {{\text{R}}_{{\text{p}}} \cap {\text{R}}_{{\text{a}}} } \right)/\left( {{\text{R}}_{{\text{a}}} } \right)$$

where ${\text{R}}_{{\text{p}}}$, ${\text{R}}_{{\text{a}}}$, and ${\text{R}}_{{\text{n}}}$ demonstrate the predicted tumor regions, actual labels, and actual non-tumor labels, respectively.

### Experimental results

To have a clear understanding and for quantitative and qualitative comparison purposes, we also implemented five other models (Multi-Cascaded^34^, Cascaded random forests^10^, Cross-modality^22^, Task Structure^21^, and One-Pass Multi-Task^23^) to evaluate the tumor segmentation performance. Quantitative results of different kinds of our proposed structure are presented in Table 1.

**Table 1:** Evaluation results with different pipeline configurations on BRATS 2018 dataset.

Method	Dice score (mean)			Sensitivity (mean)		
	Enh	Whole	Core	Enh	Whole	Core
Two-route CNN	0.2531	0.2796	0.2143	0.2456	0.2569	0.2007
Global route CNN + Attention mechanism	0.3128	0.3410	0.3025	0.3343	0.2947	0.2896
Local route CNN + Attention mechanism	0.3412	0.3671	0.3625	0.3356	0.3819	0.3808
Two-route CNN + Attention mechanism	0.4136	0.3754	0.3988	0.3910	0.3951	0.3822
Global route CNN + Preprocessing	0.7868	0.7916	0.7867	0.7426	0.7965	0.7448
Local route CNN + Preprocessing	0.8602	0.8343	0.8516	0.8751	0.8569	0.8485
Two-route CNN + Preprocessing	0.8756	0.8550	0.8715	0.8941	0.9036	0.8512
Proposed method	0.9113	0.9203	0.8726	0.9217	0.9386	0.9712

From Table 1, we can observe that the two-route CNN model without using a preprocessing approach is not able to segment the tumor area properly. Adding an attention mechanism to a two-route model without using the preprocessing method causes to gain better segmentation results in terms of all three criteria. Also, by adding the preprocessing approach, the Dice scores in three tumor regions observe a surge increase from 0.2531, 0.2796, and 0.2143 to 0.8756, 0.8550, and 0.8715 for End, Whole, and Core, respectively. Despite only having a one-route CNN model (local or Global features) and thanks to the use of the preprocessing approach, the CNN model consistently obtains improved segmentation performance in all tumor regions. Moreover, it is observed that the use of the preprocessing method is more influential than only using an attention mechanism. In other words, the proposed attention mechanism can be more helpful when we are dealing with a smaller part of the input image extracted by the preprocessing method. By comparing the effect of local and global features, it can be recognized that the local features are more effective than global features.

The Dice, Sensitivity, and HAUSDORFF99 values of all input images using all the structures are described in Table 2. For each index in Table 2, the highest Dice, Sensitivity, and the smallest HAUSDORFF99 values are highlighted in bold. From Table 2, it is obvious that our strategy can achieve the highest Sensitivity values in Enh and Whole tumor areas and the highest value for the Core area was obtained by^10^. Also, there is a minimum difference between the values of HAUSDORFF99 using^34^ and^23^. In^22^, there is a significant improvement in the Enh area for all three measures. Also^21^, achieves the worst results in the Whole and Core areas for HAUSDORFF99 measure.

**Table 2:** Comparison between the proposed method and other baseline approaches on BRATS 2018 dataset.

Method	Dice score (mean)			Sensitivity (mean)			HAUSDORFF99 (mm)		
	Enh	Whole	Core	Enh	Whole	Core	Enh	Whole	Core
^34^ Multi-Cascaded	0.7178	0.8824	0.7481	0.8684	0.7621	**0.9947**	2.80	4.48	7.07
^10^ Cascaded random forests	0.75	0.86	0.79	0.83	0.91	0.86	–	–	–
^22^ Cross–modality	0.903	0.791	0.836	0.919	0.846	0.835	4.998	3.992	6.369
^21^ Task Structure	0.782	0.896	0.824	–	–	–	3.567	5.733	9.270
^23^ One-Pass Multi-Task	0.811	0.908	0.857	–	–	–	2.881	4.884	6.932
Proposed method	0.9113	0.9203	0.8726	0.9217	0.9386	0.9712	1.669	1.427	2.408

Notice that when using the proposed method, all criteria were improved in comparison to other mentioned approaches, but the sensitivity value in the Core area using^34^ is still higher. To our best knowledge, there are three reasons. First, the proposed strategy pays special attention to removing insignificant regions inside the four modalities before applying them to the CNN model. Second, our method uses both the local and global features with different numbers of convolutional layers which explores the richer context tumor segmentation. Third, by considering the effect of the dissimilarity between the center of the tumor and the expected area, the network can be biased to a proper output class. Additionally, compared to the state-of-the-art algorithms with heavy networks, such as^22^ and^23^, our approach obtains more promising performance and decreases the running time by only using a simple CNN structure. Moreover, as shown in Table 3, the proposed method is faster at segmenting the tumor than other compared models.

**Table 3:** Comparison of execution time of different techniques applied on BRATS 2018 dataset for one subject patient.

Approach	Multi-Cascaded ^34^	Cascaded random forests ^10^	Cross-modality ^22^	Task Structure ^21^	One-Pass Multi-Task ^23^	Proposed method
Time	261 s	314 s	208 s	193 s	277 s	**84 s**

**Figure 13:**
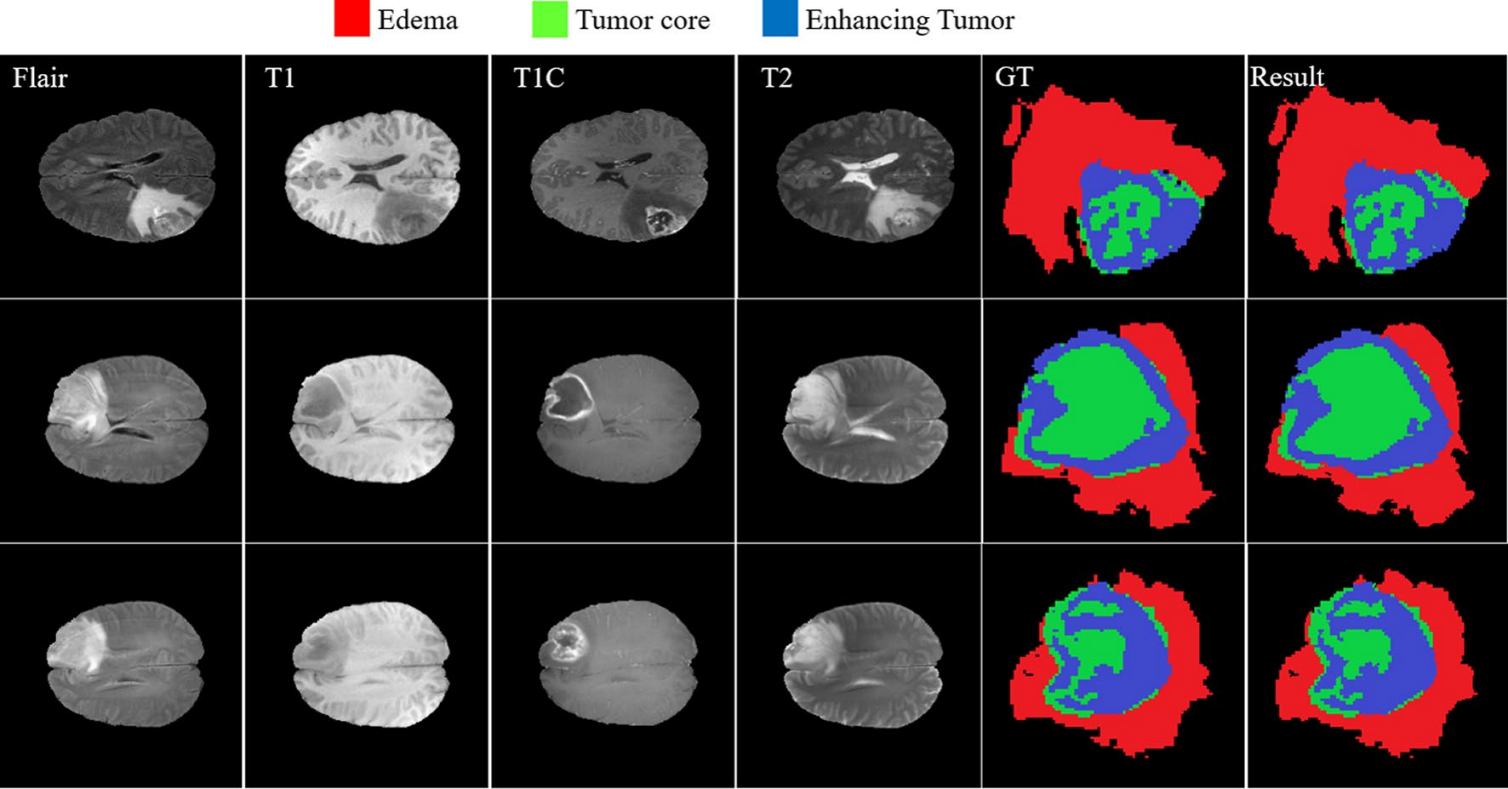
The results of brain tumor segmentation using the proposed strategy (the blue, green, and red colors are enhanced, core, and edema regions respectively).

Figure 13 provides a visual demonstration of the good results achieved by our approach on the BRATS 2018 dataset. As shown, all regions have a mutual border with all of the other regions. Due to the difference between the value of tumor core and enhancing areas inside the T1C images (third column), the border between them can be easily distinguished with a high rate of accuracy without using other modalities. But it is not true when we are dealing with the border of a tumor core, edema areas, or enhanced edema areas. Due to these mentioned characteristics of each modality, we observe that there is no need for a very deep CNN model if we decrease the searching area.

**Figure 14:**
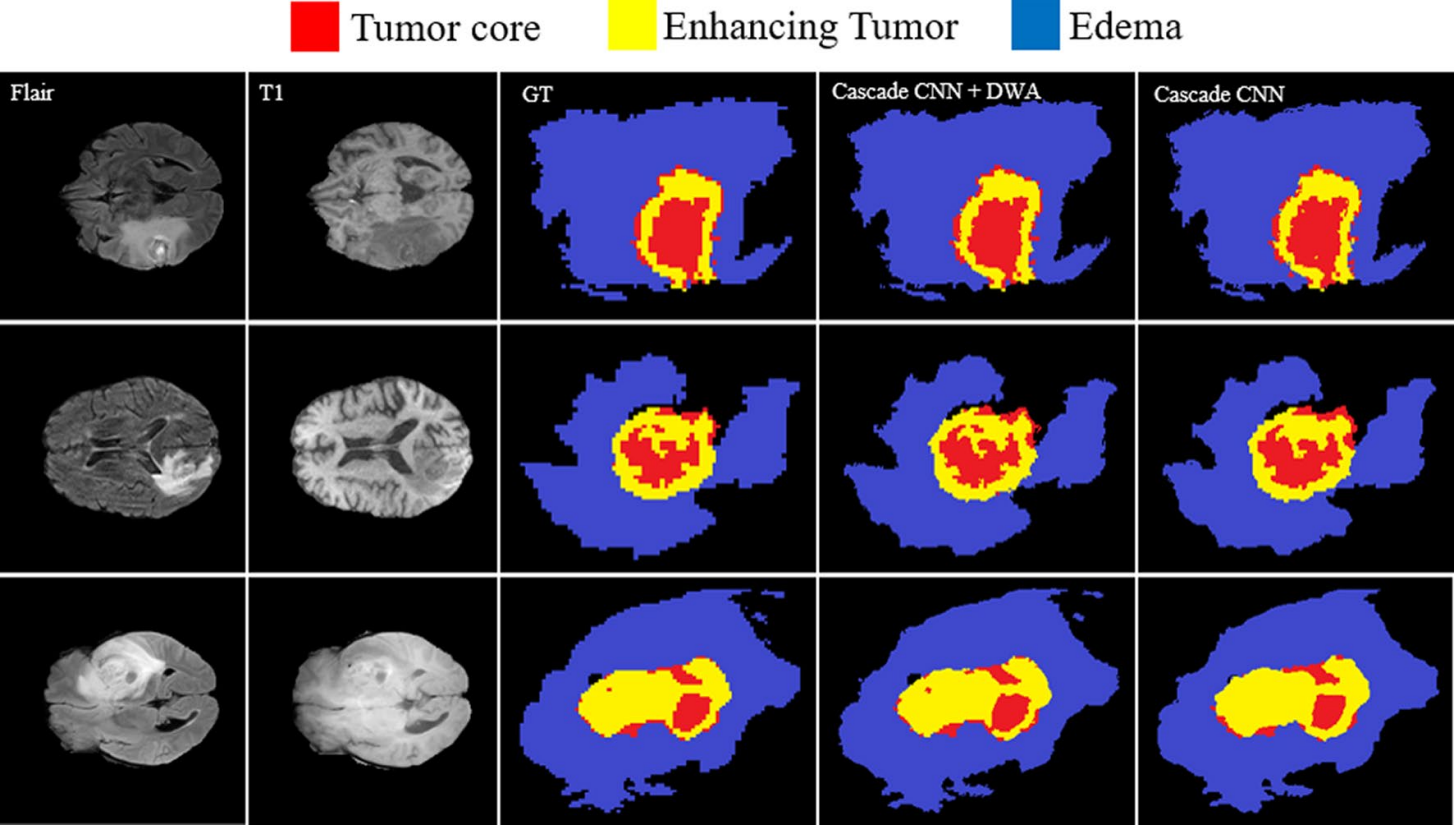
Comparing the results of brain tumor segmentation by applying DWA method to the proposed CNN structure. The blue, yellow, and red colors are edema, enhanced, and core regions respectively.

Owing to the use of the DWA module, our model can mine more unique contextual information from the tumor and the brain which leads to a better segmentation result. Figure 14 shows the improved segmentation resulting from the application of the DWA module in the proposed method—particularly in the border of touching tumor areas.

**Figure 15:**
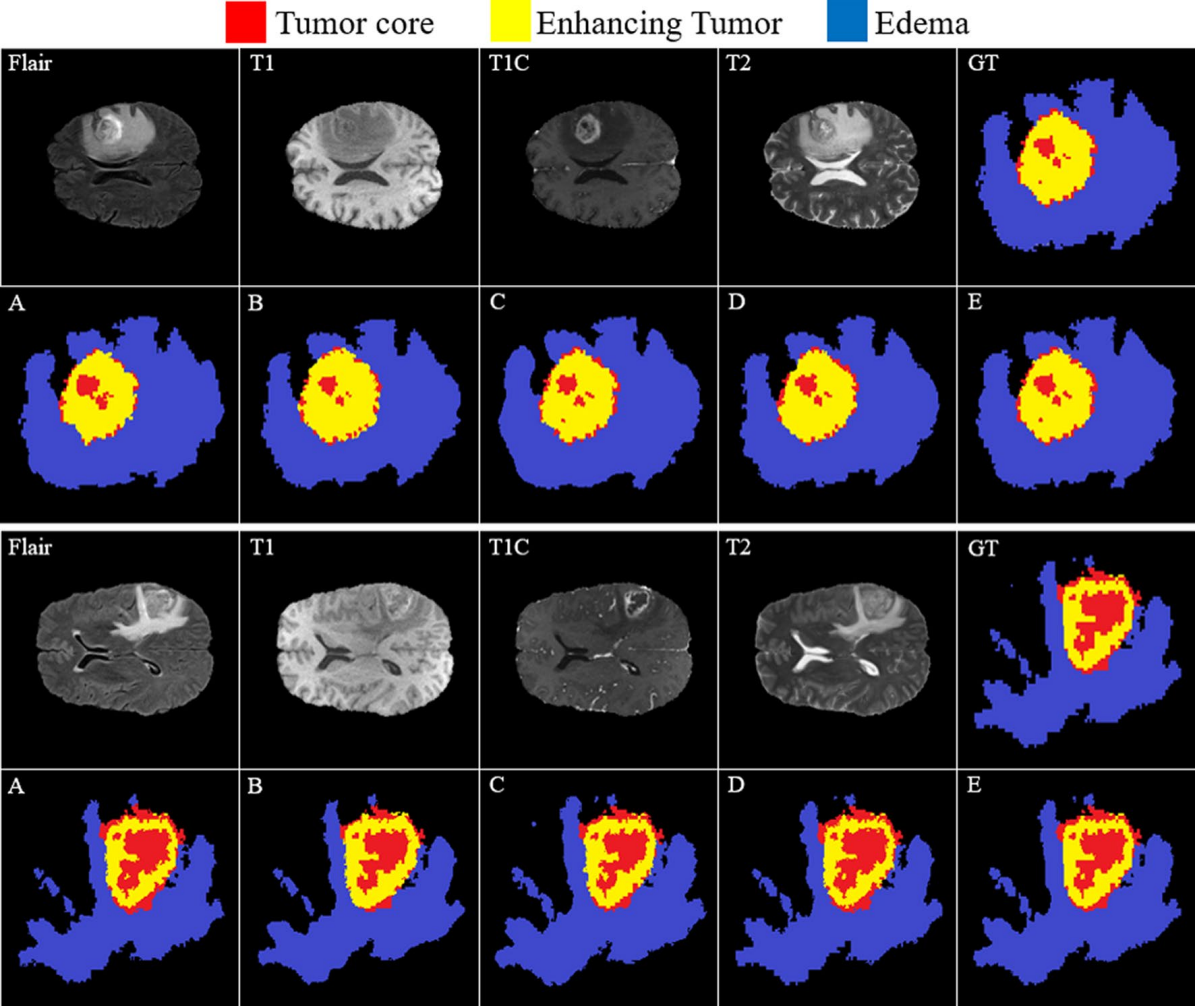
Comparing the results of brain tumor segmentation using the proposed strategy with four state-of-art methods (the blue, yellow, and red colors are edema, enhanced, and core regions respectively). (**A**) Multi-Cascaded ^34^, (**B**) Cascaded random forests ^10^, (**C**) Cross-modality ^22^, (**D**) One-Pass Multi-Task ^23^, and (**E**) Our method.

The comparison between the baseline and our model in Fig. 15 shows the effectiveness of the proposed method in the capability of distinction between all four regions.

Figure 15(GT) indicates the ground truth corresponding to all four modalities in the same row. The Multi-Cascaded (Fig. 15A) and Cascaded random forests (Fig. 15B) approaches show satisfactory results in detecting the Edema area but cannot detect the small regions of Edema outside the main Edema body. The Cross-modality (Fig. 15C) and One-Pass Multi-Task (Fig. 15D) approaches gain promising results in detecting the tumor Core and Enhancing areas, especially in detecting tumor Core in outside border of the Enhancing area.

It is illustrated that some separated Edema regions are stuck together in final segmentation using the Cross-modality method. As shown in Fig. 15(C), applying the Cross-modality structure reaches the minimum segmentation accuracy for detecting the Edema regions compared to others. This model under-segments the tumor Core areas and over-segments the Edema areas. The One-Pass Multi-Task approach shows a better core matching with the ground-truth compared to Fig. 15(A–C) but still has insufficient accuracy, especially in the Edema areas. Based on our evaluation, estimation of the three distinct regions of the brain tumor using an attention-based mechanism is an effective way to help specialists and doctors to evaluate the tumor stages which is of high interest in computer-aided diagnosis systems.

## Discussion and conclusions

In this paper, we have developed a new brain tumor segmentation architecture that benefits from the characterization of the four MRI modalities. It means that each modality has unique characteristics to help the network efficiently distinguish between classes. We have demonstrated that working only on a part of the brain image near the tumor tissue allows a CNN model (that is the most popular deep learning architecture) to reach performance close to human observers. Moreover, a simple but efficient cascade CNN model has been proposed to extract both local and global features in two different ways with different sizes of extraction patches. In our method, after extracting the tumor’s expected area using a powerful preprocessing approach, those patches are selected to feed the network that their center is located inside this area. This leads to reducing the computational time and capability to make predictions fast for classifying the clinical image as it removes a large number of insignificant pixels off the image in the preprocessing step. Comprehensive experiments have indicated the effectiveness of the Distance-Wise Attention mechanism in our algorithm as well as the remarkable capacity of our entire model when compared with the state-of-the-art approaches.

Although the proposed approach’s outstanding results compared to the other recently published models, our algorithm has still limitations when encountering tumor volume of more than one-third of the whole of the brain. This is because of an increase in the size of the tumor’s expected area which leads to a decrease in the feature extraction performance.
